# Assessment of Prescribing Practices and Factors Related to Antibiotic Prescribing in Community Pharmacies

**DOI:** 10.3390/medicina59050843

**Published:** 2023-04-27

**Authors:** Syed Arman Rabbani, Sathvik B. Sridhar, Maryam Safdar, Padma G. M. Rao, Ammar Ali Saleh Jaber, Mohammad M. AlAhmad, Khaled Shaar, Israa Emad, Muhammad Abdul Azim

**Affiliations:** 1 Department of Clinical Pharmacy and Pharmacology, RAK College of Pharmacy, RAK Medical and Health Sciences University, Ras Al Khaimah P.O. Box 11172, United Arab Emirates; 2 Dubai Pharmacy College for Girls, Dubai P.O. Box 19099, United Arab Emirates; 3 Department of Clinical Pharmacy, College of Pharmacy, Al Ain University, Abu Dhabi P.O. Box 64141, United Arab Emirates

**Keywords:** antibiotics, antimicrobial resistance, prescribing practices, community pharmacies, United Arab Emirates

## Abstract

*Background and Objectives*: Overprescribing of antibiotics is one of the important contributors of antimicrobial resistance globally. A high proportion of antibiotics prescribed in community settings are unnecessary or inappropriate. This study assesses the prescribing practices and factors related to antibiotic prescribing in community pharmacies in United Arab Emirates (UAE). *Materials and Methods*: A cross-sectional study utilizing a quantitative approach was carried out in the community pharmacies of Ras Al Khaimah (RAK), UAE. Six hundred and thirty prescription encounters from 21 randomly selected community pharmacies were investigated using World Health Organization (WHO) core prescribing indicators. Factors related to antibiotic prescribing were identified using logistic regression analyses. *Results*: In 630 prescription encounters, a total of 1814 drugs were prescribed. Out of these, the most commonly prescribed drug class was antibiotics (43.8% prescriptions) and the antibiotic was amoxicillin/clavulanic-acid (22.4%). The average number of drugs per prescription was 2.88, which was higher than the WHO recommended value of 1.6–1.8. In addition, more than half of the prescriptions (58.6%) had drugs by generic names and the majority of the drugs prescribed (83.8%) were from the essential drug list, which were lower than the optimal values of 100%. The majority of the antibiotics prescribed in the study were from the WHO’s Access group antibiotics. Multivariable logistic regression analysis identified patient age (children—OR: 7.40, 95% CI: 2.32–23.62, *p* = 0.001 and adolescent—OR: 5.86, 95% CI: 1.57–21.86, *p* = 0.008), prescriber qualification as general practitioner (OR: 1.84, 95% CI:1.30–2.60, *p* = 0.001), and number of drugs per prescription (OR: 3.51, 95% CI: 1.98–6.21, *p* < 0.001) as independent factors associated with antibiotic prescribing. *Conclusions*: This study reveals considerable variations from the WHO recommendations for the different prescribing indicators in the community pharmacies of RAK, UAE. In addition, the study reports overprescribing of antibiotics in the community setting, indicating the need for interventions to promote rational use of antibiotics in a community setting.

## 1. Introduction

Community pharmacies play a vital role in the provision of pharmaceutical care services to patients in the community. These pharmacies are accessible and convenient for patients, which makes them an indispensable part of the healthcare system [1,2]. Patients rely on community pharmacies for their medication needs, and the pharmacists in these facilities are often the first point of contact for patients seeking advice and information on their diseases and medications [3]. Community pharmacies provide a wide range of pharmaceutical care services, such as dispensing prescription, over-the-counter and herbal medications, giving medication counseling, monitoring drug therapy, and identifying and resolving drug-related problems [1,2,4]. Furthermore, community pharmacies contribute to health screenings, immunizations, and other preventive health services [4]. The significance of community pharmacies in providing pharmaceutical care services is widely recognized, and their role is expanding rapidly as the demand for healthcare services increases [5,6].

Rational use of medicines is essential as it ensures patient safety, optimizes therapeutic outcomes, prevents the development of drug-related problems, and reduces the overall cost of healthcare [7]. The World Health Organization (WHO) defines rational use of medication as the provision of “right medicine to the right patient, in the right dose, for the right duration of time, at the most economical cost to the patients and their community” [8]. According to the WHO, it is estimated that over half of all medicines are not properly prescribed, dispensed, or taken, leading to adverse patient outcomes [9]. Irrational use of medications has become a global problem. This problem includes practices such as prescribing medications by brand names, polypharmacy, overprescribing of antibiotics, and excessive use of injections among others [10]. Periodic assessment of prescribing and dispensing practices helps in checking and improving upon these practices. In addition, this assessment can help in providing feedback to the healthcare providers, leading to more appropriate and effective medication use.

Overprescribing of antibiotics in primary care is one of the important contributors of antimicrobial resistance (AMR) globally [11]. Previous studies estimated that a high proportion of antibiotics prescribed in community settings are unnecessary or inappropriate [12,13]. The first step in checking the inappropriate prescribing and dispensing of antibiotics in community settings is to periodically assess the antibiotic utilization patterns and compare these patterns with the quality indicators. WHO has developed core drug use indicators to assess the performance of healthcare facilities, including prescribing indicators, patient care indicators, and health facility indicators [14]. These indicators provide a comprehensive framework for monitoring drug use patterns, in general, and of antibiotics in particular, improving quality of care and evaluating health system performance, including the provision of pharmaceutical care services. Furthermore, these quality indicators could serve as a benchmark for the introduction of antibiotic stewardship in the community settings [7].

Many studies have assessed general and antibiotic specific prescribing practices employing WHO core indicators in different settings, community pharmacies [15,16,17,18,19,20,21], primary health care centers [22,23], general [24] and referral [9] hospitals. The majority of these studies reported deviation from the optimum values of WHO core indicators, highlighting the need for regular review of drug use practices, education and training for health care providers on responsible drug use, and fostering a culture of continuous quality improvement. However, only a few studies have assessed the prescribing practices and antibiotic prescribing in United Arab Emirates (UAE) using these drug use indicators [25,26]. This study aims to assess the prescribing practices and factors related to antibiotic prescribing in community pharmacies of UAE.

## 2. Materials and Methods

### 2.1. Study Design and Setting

This descriptive cross-sectional study was carried out in the community pharmacies of RAK, which is one of the seven emirates of UAE and is situated in the northernmost part of the country with an estimated population of 345,000 [27]. The study was conducted over a period of three months (February 2022 to April 2022).

The UAE has a rapidly developing and high-quality healthcare system, with a mix of public and private healthcare providers serving a diverse multiethnic population. Ministry of Health and Prevention (MOHAP) is responsible for regulating healthcare services in the country and has implemented several initiatives aimed at improving healthcare quality and patient safety. According to UAE regulations, antibiotics are prescription-only medications and require a valid prescription from a registered medical practitioner. The MOHAP has launched several campaigns and initiatives aimed at promoting appropriate antibiotic use and preventing the emergence of antibiotic resistance [28].

### 2.2. Study Population and Sample Size

We stratified the community pharmacies located in RAK into five main geographical areas and randomly selected a sample of four or more pharmacies from each of these areas, taking into consideration the number of pharmacies present and the willingness of the community pharmacists to take part in the study. Following this stratified random sampling, 21 pharmacies were included in the study. According to the WHO, the minimum number of encounters required for prescription analysis is 600. Therefore, in the present study, 630 prescriptions were analyzed, with 30 prescriptions being selected randomly from each pharmacy. Prescriptions written by diverse medical professionals, including general practitioners and specialists, were collected and analyzed. Specialists were medical practitioners, such as physicians, pediatricians, gynecologists, cardiologists, dermatologists, ear, nose, and throat specialists, ophthalmologists, internal medicine specialists, and general surgeons.

### 2.3. Data Collection and Analysis

Study investigators collected data related to prescribing indicators in accordance with the WHO guidelines and methods. Data related to prescribing indicators included ‘average number of drugs per encounter, percentage of drugs prescribed by generic name, percentage of encounters with an antibiotic prescribed, percentage of encounters with an injection prescribed, percentage of drugs prescribed from essential drugs list or formulary’. In addition, data related to patient age and gender, prescriber qualification, pharmacist-related information, such as age, gender, qualification, work experience, position in pharmacy, type of pharmacy, and location of pharmacy, were collected.

IBM^®^ SPSS^®^ Statistics Software version 27.0 was used for data analysis. Before analyzing the data, the skewness and kurtosis were assessed. Data normality was confirmed by the Shapiro–Wilk test. Categorical variables were represented as frequency and percentage with 95% confidence intervals (CI). Continuous variables were represented as median and interquartile range with 95% CI. Chi square test or Fisher’s exact test was used for comparing categorical variables, while the two-sample median test was used for continuous variables. Factors related to antibiotic prescribing were identified using logistic regression analyses. The results were presented as odds ratios (OR) along with 95% CI. Significance level (α) was 0.05 for all statistical tests.

### 2.4. Ethical Approval

The Ministry of Health and Prevention Research and Ethics Committee/RAK Subcommittee (Approval No. MOHAP/REC/2022/2-2022-UG-D) and Ras Al Khaimah Medical and Health Sciences University Research and Ethics Committee (Approval No. RAKMHSU-REC-016-2021/22-UG-D) approved the study protocol.

## 3. Results

### 3.1. Characteristics of Patients, Pharmacists, and Prescriptions

Six hundred and thirty prescriptions were evaluated, belonging to 630 patients, of which 54.3% (*n* = 342) were female and 45.7% (*n* = 288) were male with a median age of 29 years (IQR: 14–42; 95% CI: 27–32). Regarding the pharmacists in the community pharmacies, the majority were male (*n* = 15; 71.4%) with a median experience of 5.0 years (IQR: 4.0–7.0), had a bachelor’s degree in pharmacy (*n* = 13; 61.9%), had a designation as a pharmacist (*n* = 12; 57.1%), received less than 100 prescriptions per day (*n* = 19; 90.5%), and worked in independent type of pharmacies (*n* = 15; 71.4%). The majority of the study prescriptions were written by general practitioners (*n* = 365; 57.9%), followed by gynecologists (*n* = 49; 7.8%) and pediatricians (*n* = 35; 5.6%). Table 1 represents the characteristics of study patients, prescriptions, and pharmacists.

### 3.2. Prescribing Practices

**Figure 1:**
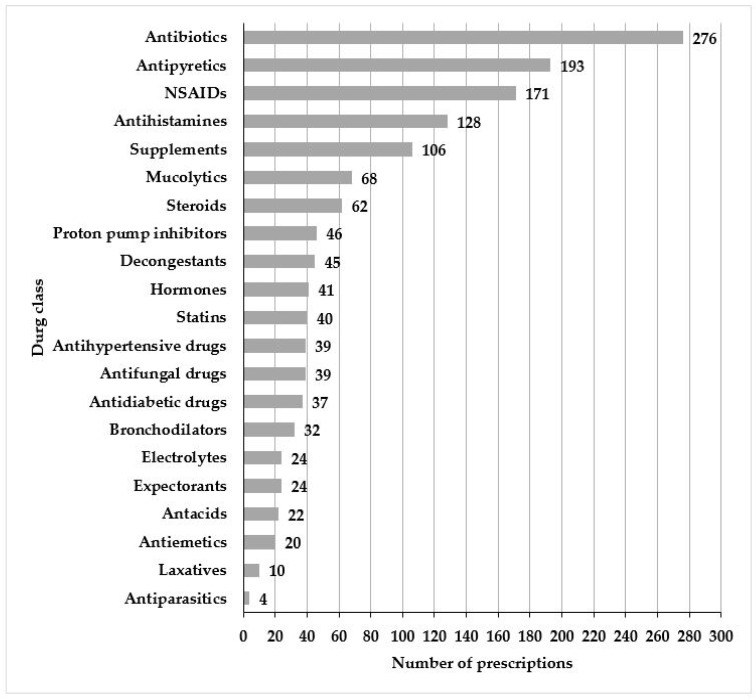
Drug classes prescribed in the community pharmacies.

In 630 prescription encounters, a total of 1814 drugs were prescribed. Out of these, the most commonly prescribed drug class was antibiotics (43.8% prescriptions), followed by antipyretics (30.6% prescriptions), non-steroidal anti-inflammatory drugs (27.1% prescriptions), and antihistamines (20.3% prescriptions). Among antibiotics, the commonly prescribed antibiotics were amoxicillin/clavulanic-acid (22.4%), azithromycin (4.4%), and cefixime (4.1%) (Figure 1). The majority of the encounters had two drugs per prescription (*n* = 174, 27.6%), had at least one drug prescribed by generic name (*n* = 369, 58.6%), had no antibiotic encounter (*n* = 354, 56.2%), had no encounter with injection (*n* = 627, 99.5%), and had drugs from the essential drug list (*n* = 529, 84%). Table 2 represents the distribution of different prescribing indicators.

### 3.3. WHO Prescribing Indicators

The average number of drugs per prescription was 2.88, with antibiotics prescribed in 43.8% of the prescriptions, which were higher than the optimal values of 1.6–1.8 and 20.0–26.8%, respectively. More than half of the prescriptions (58.6%) had drugs by generic names and the majority of the drugs prescribed (83.8%) were from the essential drug list, which were lower than the optimal values of 100%. Furthermore, only three prescriptions had injections, accounting for 0.5% of all the encounters. Table 3 represents a comparison of the WHO prescription indicators in the current study and other studies conducted in community pharmacies in different parts of the world.

### 3.4. Antibiotic Prescribing

Prescribed antibiotics were categorized into three groups, Access, Watch, and Reserve, as per the WHO’s AWaRe classification [28]. The majority of the antibiotics (163/276) prescribed in the study were from the Access group, with amoxicillin/clavulanic-acid being prescribed the most (22.4%), followed by amoxicillin (3.3%) and sulfadiazine (0.2%). Commonly prescribed antibiotics from the Watch group were azithromycin (4.4%), cefixime (4.1%), cefuroxime (3.2%), ciprofloxacin (2.2%), fusidic acid (1.4%), while none of the antibiotics were prescribed from the Reserve group (Table 4).

### 3.5. Factors Related with Antibiotic Prescribing

To identify the factors related to antibiotic prescribing, a univariable logistic regression analysis, including patient age, patient gender, prescriber qualification, and number of drugs per prescription as independent variables, was performed. Patient age (standard age ranges) and number of drugs per prescription were converted into categorical variables before putting them into the model. The analysis revealed that children and adolescent patients were more likely to be prescribed antibiotics (OR: 7.9, 95% CI: 2.5–24.65, *p* < 0.001; OR: 5.3, 95% CI: 1.4–18.95, *p* = 0.010, respectively) compared to older patients. In addition, general practitioners were more likely to prescribe antibiotics (OR: 1.71, 95% CI: 1.23–2.36, *p* = 0.001) compared to specialists. Moreover, prescriptions with a greater number of drugs were more likely to have antibiotics (OR: 3.51, 95% CI: 1.98–6.21, *p* < 0.001). Multivariable logistic regression analysis identified patient age (children—OR: 7.40, 95% CI: 2.32–23.62, *p* = 0.001 and adolescent—OR: 5.86, 9%% CI: 1.57–21.86, *p* = 0.008) as the independent predictors of antibiotic prescribing in our study population. Furthermore, prescriber qualification as general practitioner (OR: 1.84, 95% CI: 1.30–2.60, *p* = 0.001) and number of drugs per prescription (OR: 3.51, 95% CI: 1.98–6.21, *p* < 0.001) were independently associated with antibiotic prescribing (Table 5).

## 4. Discussion

Assessing prescribing patterns, in general and of antibiotics, can provide valuable insights into prescribing practices and identify areas for improvement and promote rational drug use. Currently, studying antibiotics use in a community setting is an important area of focus in healthcare due to the growing concern of antibiotic resistance. WHO indicators provide a comprehensive framework for assessing this drug use. The average number of drugs per encounter in this study was higher than the WHO optimal values and the values reported in the community pharmacies of Saudi Arabia [22], Brazil [21], Eritrea [17], and Nepal [19]. However, it was lower than the values reported in the community pharmacies of India [18,29] and Pakistan [20]. Higher number of drugs per prescription indicates overprescribing, which can lead to negative patient outcomes, such as a higher risk of adverse drug reactions, medication errors, and lower medication adherence [30].

Generic prescribing is important because it promotes affordability, accessibility, and equity in healthcare delivery. WHO recommends that 100% of drugs should be prescribed by generic names [7]. However, in this study, only 58.6% of the medications were prescribed by their generic names, which is significantly lower than the WHO standards. Similar findings were reported by studies conducted in Bahrain [31], Saudi Arabia [22], Brazil [21], Nepal [23], and African regions [32] where much of the prescribing was by brand names.

In this study, the percentage of encounters with antibiotics (43.8%) was significantly higher than the WHO recommendation of 20–26.2% [7] pointing towards overprescribing of antibiotics. A high number of studies conducted in the community settings in different parts of the world have reported similar overprescribing of antibiotics, with the values ranging from 32.2% to 58.8% [15,16,17,18,19,22,23,33,34]. This inappropriate antibiotic prescribing in community settings can have serious implications for public health, including the development of antimicrobial resistance, increased healthcare costs, adverse drug reactions, disruption of the microbiome, and increased medicalization of self-limiting conditions [30,35]. To address this issue, it is important to promote appropriate use of antibiotics, to educate healthcare providers and patients about the risks of overuse, and most importantly, to develop and implement antimicrobial stewardship programs with a focus on community/primary care settings.

The WHO AWaRe classification system was developed to promote responsible prescribing behavior by increasing the use of antibiotics in the Access group and decreasing the use of antibiotics in the Watch and Reserve groups [36]. According to the WHO, if more than 60% of all antibiotics are prescribed from the Access group, it would ensure the availability of essential antibiotics, lower the risk of antibiotic resistance, and enhance responsible antibiotic use [28]. In this study, the majority of antibiotics (60%) were prescribed from the Access group, which is noteworthy since it is line with the WHO recommendations [28]. The antibiotics prescribed from the Access group were amoxicillin/clavulanic-acid followed by amoxicillin and sulfadiazine. Antibiotics prescribed from the Watch group were approximately 40%, which is relatively higher than contemporary studies [17]. This could be due to the fact that the majority of the patients in this study were outpatients. Furthermore, no antibiotic was prescribed from the Reserve group, which is consistent with previous studies [17,37] and demonstrates responsible prescribing of antibiotics.

Previous studies have reported different factors associated with antibiotic prescribing, such as type of infection, antibiotic sensitivity tests, antibiotic availability in health facilities, prescriber’s qualification, knowledge, training, and experience, source of payment to prescribers, socio-economic status of patients, age of patients, etc. [38,39,40]. Patient age, prescriber qualification, and number of drugs per prescriptions were found to be the independent factors associated with antibiotic prescribing in the current study. Antibiotic prescriptions were significantly associated with age of patients, with children and adolescents being more likely to receive antibiotic prescriptions. These results are similar to studies carried out in other countries [38,41,42,43] where high rates of antibiotic prescriptions were found among the pediatric population. These findings can be attributed to the diagnostic uncertainty and prognostic considerations that go into antibiotic prescribing in children. Parental anxiety and time constraints also affect this decision-making process. Clinicians may be more inclined to prescribe antibiotics when they feel pressure from parents. The relatively young age of the study patients (29 years [14,15,16,17,18,19,20,21,22,23,24,25,26,27,28,29,30,31,32,33,34,35,36,37,38,39,40,41,42]) can be attributed to the demographic profile of the UAE, where the majority of the population is younger than 40 years old [44], and to the community setting of the study, which is the first point of contact for younger patients seeking treatment for common diseases [45].

In this study, prescriber qualification was significantly associated with antibiotic prescribing, with general practitioners being more likely to prescribe antibiotics compared to specialists. This finding is in line with previous studies conducted in the United States [46] and Germany [47], where general practitioners more frequently prescribed antibiotics than medical specialists. This could be due to several factors, including diagnostic uncertainties, lack of training and information on antibiotic prescribing, pressure from patients to prescribe antibiotics, and a lack of time to fully evaluate patients’ conditions.

This study has some limitations. The study has limited generalizability, as it was conducted in a specific community pharmacy setting. The sample of community pharmacies and pharmacists included in the study may not be representative of all community pharmacies or pharmacists in the UAE. The cross-sectional design of the study limits causal conclusions, as it only captured a snapshot of prescribing practices and factors related to antibiotic prescribing at a single point in time. The unavailability of indication data for the prescribed antibiotics in the community pharmacies restricted the assessment of appropriateness of the antibiotics. The study did not take self-medication into consideration, which might have underestimated the antibiotic use in the study population. The study recorded only the number of co-prescribed medications with antibiotics and did not analyze specifically the individual co-prescribed drugs.

## 5. Conclusions

This study reveals considerable variations from the WHO recommendations for the different prescribing indicators in community pharmacies. In addition, the study reports overprescribing of antibiotics in the community setting. However, the majority of the antibiotic prescriptions were from the WHO’s Access group antibiotics. Patient age and prescriber qualification were found to be the independent factors associated with antibiotic prescribing. The results of this study indicate the need for interventions to promote appropriate use of antibiotics, to educate healthcare providers and patients about the risks of overuse, and most importantly, to develop and implement antimicrobial stewardship programs that specifically focus on community and primary care settings.

## Figures and Tables

**Table 1:** Characteristics of study patients, prescriptions, and pharmacists.

Variable	N (%)/Median (IQR)	95% CI
**Patient characteristics** (N = 630)		
**Age (years)**	29 (14–42)	27–32
Infants: <1	13 (2.1)	1.1–3.3
Children: 1–12	135 (21.4)	18.1–24.8
Adolescents: 13–17	33 (5.2)	3.3–7.0
Adults: 18–64	425 (67.5)	63.8–71.4
Old adults: ≥65	24 (3.8)	2.5–5.4
**Gender**		
Male	288 (45.7)	41.9–49.5
Female	342 (54.3)	50.5–58.1
**Prescription characteristics** (N = 630)		
**Prescriber specialty**		
General practitioner	365 (57.9)	54.1–62.1
Gynecologist	49 (7.8)	5.7–10.0
Pediatrician	35 (5.6)	3.7–7.3
Cardiologist	22 (3.5)	2.2–4.9
Ophthalmologist	14 (2.2)	1.1–3.5
Endocrinologist	3 (0.5)	0.0–1.1
General surgeon	10 (1.6)	0.6–2.5
Dentist	27 (4.3)	2.7–6.0
Dermatologist	25 (4.0)	2.4–5.4
ENT specialist	50 (7.9)	5.9–10.2
Internal medicine specialist	30 (4.8)	3.2–6.5
**Pharmacist characteristics** (N = 21)		
**Age (years)**	32 (30–34)	31.5–36.0
**Gender**		
Male	15 (71.4)	52.4–90.5
Female	6.0 (28.6)	9.5–47.6
**Education**		
BPharm	13.0 (61.9)	42.9–81.0
PharmD	5.0 (23.8)	4.8–42.9
Master’ degree	3.0 (14.3)	0.0–28.6
**Work Experience**	5.0 (4.0–7.0)	4.0–7.0
**Position in pharmacy**		
Pharmacist In charge	6.0 (28.6)	14.3–47.6
Pharmacist	12.0 (57.1)	33.3–76.2
Assistant Pharmacist	3.0 (14.3)	0.0–28.6
**Type of pharmacy**		
Independent	15.0 (71.4)	52.4–90.5
Chain	6.0 (28.6)	9.5–47.6
**Number of prescriptions per day**		
<100	19 (90.5)	76.2–100
100–150	2.0 (9.5)	0.0–23.8

CI: confidence interval, IQR: Interquartile range.

**Table 2:** Distribution of prescribing indicators.

Prescribing Indicators	N (%)	95% CI
**Number of drugs per prescription**		
One	137 (21.7)	18.4–25.1
Two	174 (27.6)	24.0–31.3
Three	146 (23.2)	20.0–26.3
Four	68 (10.8)	8.6–13.3
Five or more	105 (16.7)	13.8–19.5
**Number of drugs prescribed by generic name**		
None	261 (41.4)	37.6–45.4
One	130 (20.6)	17.3–23.8
Two	83 (13.2)	10.8–15.7
Three	61 (9.7)	10.8–15.7
Four	30 (4.8)	3.0–6.5
Five or more	65 (10.3)	8.3–12.9
**Number of drug encounter with antibiotics**		
None	354 (56.2)	52.4–60.0
One	236 (37.5)	33.7–41.4
Two	39 (6.2)	4.1–8.3
Three	1 (0.2)	0.0–0.5
**Number of drug encounter with injection**		
None	627 (99.5)	98.9–100
One	3 (0.5)	0.0–1.1
**Number of drugs prescribed from essential drug list**	529 (84)	80.8–86.7

CI: confidence interval.

**Table 3:** Comparison of WHO prescription indicators in the current study and other studies conducted in community pharmacies.

Prescribing Indicators	Optimal Value	Current Study	Bassoum et al. [2]	Jacob et al. [3]	Amaha et al. [4]	Aravamuthan et al. [5]	Chapagain et al. [6]	Atif et al. [7]	Vooss et al. [8]	Mahalli et al. [9]
Average number of drugs per prescription	1.6–1.8	2.88	2.5	2.89	1.76	3.7	2.14	4.5	2.03	2.4
Percentage of drugs prescribed by generic name	100%	58.6	7.0	0.75	83.14	8.0	45.18	23.3	72.8	61.2
Percentage of prescriptions with an antibiotic prescribed	20.0–26.8%	43.8	40	37.8	53	58.8	40.44	39.6	21.7	32.2
Percentage of encounters with an injection prescribed	13.4–24.1%	0.5	7	2.74	7.8	24.3	3.44	19	2.4	2
Percentage of drugs prescribed from the list of essential drugs	100%	83.8	32	30.08	93.39	100	76.11	54.4	80.3	99.2

**Table 4:** Classification of prescribed antibiotics as per AWaRe methodology [28].

AWaRe Classification		
Access (%)	Watch (%)	Reserve (%)
Amoxicillin/clavulanic-acid (22.4)	Azithromycin (4.4)	-
Amoxicillin (3.3)	Cefixime (4.1)	
Sulfadiazine (0.2)	Cefuroxime (3.2)	
	Ciprofloxacin (2.2)	
	Fusidic acid (1.4)	
	Moxifloxacin (0.8)	
	Levofloxacin (0.5)	
	Ofloxacin (0.2)	
	Ceftriaxone (0.2)	

**Table 5:** Factors associated with antibiotic prescribing.

Variable	Antibiotic Prescribing		Logistic Regression			
	No	Yes	COR (95%CI)	*p*-Value *	AOR (95% CI)	*p*-Value *
**Patient age**						
Infants: <1	10 (1.6%)	3 (0.5%)	1.5 (0.28–8.03)	0.630	1.01 (0.18–5.60)	0.987
Children: 1–12	52 (8.3%)	83 (13.2%)	7.9 (2.5–24.65)	<0.001	7.40 (2.32–23.62)	0.001
Adolescents: 13–17	16 (2.5%)	17 (2.7%)	5.3 (1.4–18.95)	0.010	5.86 (1.57–21.86)	0.008
Adults: 18–64	256 (40.6%)	169 (26.8%)	3.3 (1.1–9.82)	0.032	2.77 (0.90–8.54)	0.075
Old adults: ≥65	20 (3.2%)	4 (0.6%)	Ref.		Ref.	
**Patient gender**						
Male	154 (24.4%)	134 (21.3%)	Ref.		Ref.	
Female	200 (31.7%)	142 (22.5%)	0.82 (0.59–1.12)	0.207	0.95 (0.68–1.34)	0.786
**Prescriber qualification**						
General practitioner	185 (29.4%)	180 (28.6%)	1.71 (1.23–2.36)	0.001	1.84 (1.30–2.60)	0.001
Specialist	169 (26.8%)	96 (15.2%)	Ref.		Ref.	
**Number of drugs per prescription**						
One	101 (16%)	36 (5.7%)	Ref.		Ref.	
Two	94 (14.9%)	80 (12.7%)	2.38 (1.47–3.87)	<0.001	2.82 (1.69–4.69)	<0.001
Three	71 (11.3%)	75 (11.9%)	2.96 (1.79–4.88)	<0.001	3.21 (1.9–5.43)	<0.001
Four	35 (5.6%)	33 (5.2%)	2.64 (1.43–4.86)	0.002	3.019 (1.59–5.73)	0.001
Five or more	53 (8.4%)	52 (8.3%)	2.75 (1.6–4.72)	<0.001	3.51 (1.98–6.21)	<0.001

COR: crude odds ratio, AOR: Adjust odds ratio, CI: confidence interval, * *p* < 0.05 was considered significant.

## Data Availability

Data will be available from the corresponding author upon reasonable request.
